# NEWS – RESOURCES

**DOI:** 10.7189/07.020203

**Published:** 2017-12

## Demography

➢India’s government plans to replace tax identification cards, known as Permanent Account Number (PAN) cards, with a biometric–based personal identification number (Aadhaar) have been dealt a blow by the country’s top court. The court ruled that tax–payers who had opted out of getting an Aadhaar number should not be forced to get one, meaning that they are not yet mandatory to file tax returns. The government argues that PAN cards are easy to fake, and that a person can get several PAN cards. However, critics say that the government cannot forcibly take people’s biometric details, and have raised concerns over the security of the world’s largest biometric database, which has already been affected by a number of leaks of citizens’ records. Over the past 8 years, the Indian government has collected fingerprints and iris scans from more than 1 billion residents – nearly 90% of the population – and stored them in a high–security digital centre, and each person was provided with a randomly–generated, unique 12–digit identity number. It has been using Aadhaar to transfer government pensions, scholarships, wages for a jobs–for–work scheme and benefits for cooking fuel to recipients, as well as to distribute cheap food to poorer people. (BBC, 6 June 2017)

➢Soybeans are likely to replace corn as the most widely sown crop in the USA, and soybeans harvests are also increasing in Brazil and Argentina. The rise of soybean harvests is due to China's trebling its soybean imports over the past 10 years, to an estimated 93 million tonnes – and imports increased even as its imports of other industrial commodities have fluctuated. The global demand for wheat (increasing 1% each year) is in line with population growth, but soybean demand has risen by 5% each year. The bulk of this growth is due to its use in animal foodstuffs – soybeans are high in protein and livestock fed on it fatten quickly – and it is the only plant protein with complete amino acids. To meet the rising demand, animal husbandry in China is changing from small–scale operations where food scraps are fed to pigs, to industrial businesses. Meat consumption in China is rapidly rising, driven by a growing urban population – increasing by 20 million every year – which tends to eat more meat. (Financial Times, 20 June 2017)

➢According to a report by UNESCO, the UN education agency, statistics on the global access to education could be wrong by up to 350 million children – equivalent to the combined populations of the UK, Germany, Frank, Italy and Spain. These “invisible” children are typically growing up as “the poorest of the poor” in places unreached by census–takers and administrators. These unregistered people may live in city slums in developing countries, or in families living illegally as migrants. Conventional means of gathering population information – eg, surveys, censuses and records – are only accurate for settled people accessing services. The report highlights how holding governments accountable for delivering education depends on knowing how many people need to be supported; and global goals for reducing illiteracy and increasing access to school must recognise that some of the world’s poorest people are not even part of the target. It also warns that education has received a decreasing share of aid budgets – when UNICEF recently reported that there has been “nearly zero progress” on improving access to school in the world’s poorest countries over the past 10 years. This is only worsened by a “staggering” problem in teaching quality, with more than 600 million young people who have attended school but lack basic literacy and numeracy skills. (Reuters, 6 September 2017)

➢By 2050, 70% of the world’s population will live in cities – including 1 billion people in China, 875 million people in India, and 365 million people in the Americas. This mass migration will mean that urban areas have to be capable of handling energy, transport, housing, economic growth etc. Moreover, large urban areas bring together people with different languages, backgrounds and cultures, leading to potential clashes, crime and terrorism. Darrell M West and Daniel Bernstein have examined how digital technology, mobile networks and integrated solutions have helped 17 cities worldwide to manage public safety and law enforcement. The cities under study range from Jakarta, Kuwait, Abuja, Bogota, London and Riyadh, and were chosen to because of geographic diversity, population size and capital city status. They found that the cities that have implemented and adopted best practice in digital technology have a clear vision, financial resources and strong infrastructures, generating positive safety outcomes. They recommend increased resources for digital infrastructure investments, implementing integrated command centres, building public support, using crowd–sourcing platforms to encourage citizen participation, breaking down organisational stovepipes through technology, using police body and CCTV cameras to improve accountability, making data openly available and deploying data analytics, and balancing privacy and security concerns. (Brookings Institute, 23 October 2017)

➢Japan is set to become the world’s first “ultra–aged” country, with more than 28% of its population aged over 65 years. Currently 27.3% of Japan’s population is aged over 65 years, and this is expected to reach 37.7% in 2050. However, poverty amongst older people is an increasing problem, and may be behind the high levels of criminal activity seen in people aged over 65 years. Most criminals in this age group are charged with petty crimes – eg, shoplifting and theft, and this may be underpinned by economic hardship experienced by people aged over 65 years. This group is also more likely to re–offend, with nearly 25% being re–arrested within 2 years of release, compared to 10% for offenders aged up to 29 years. Japan’s austere prison conditions – including no talking whilst at work, inmates being obliged to walk in single files, and restrictions on bathing – is not acting as a deterrent to the increasing re–offending rates amongst this group. The situation has become so severe that the government has approved a plan to deploy nursing care staff to about 50% of Japan’s prisons, from April onwards. (Japan Times, 18 November 2017)

## Economy

➢Since the 1990s, North Africa has suffered from competition from east Europe and Southeast Asia – both sources of cheap and skilled labour – hampering its efforts to expand its manufacturing base beyond the textiles, mechanical, electrical and cement sectors, and to become a cheap production base for European industry. However, rising wages in east Europe are encouraging companies to shift production in the labour–intensive parts of their supply chains elsewhere, and some countries, such as Morocco, have made gains in new industrial sectors. Now economists are questioning if the region can emulate the Asian “flying geese”, where industrial production shifted from mature economies such as Japan towards emerging economies (initially South Korea, Taiwan, Singapore and Hong Kong, then latterly Malaysia, Indonesia, Thailand etc), due to the product cycle, lower labour costs, and state invention. Now, North African countries are eyeing the same path, due to their low labour costs, improving physical and human capital and supportive industrial policies. These countries are politically stable, although they have high debt levels and unemployment. However, problems of low education standards and high wages in the public sector – which deter unemployed workers from taking lower–paid jobs in the private sector – could prevent an industrial boom, alongside poor infrastructure and governments which are struggling to adapt to the changing political environment. (Financial Times, 26 July 2017)

➢New research from Alan Krueger of Princeton University shows that the increasing use of opioids in the USA is impeding its jobs market recovery, as high rates of painkiller usage is linked to falling numbers of men participating in the labour market. It may account for 20% of men’s declining labour–force participation from 1999 to 2015, and labour–market participation fell most sharply in areas with higher rates of opioid prescriptions, whilst these prescriptions increased by 356% in the same period. Separate research has shown that nearly 50% of working–age men who are not in employment, nor actively seeking employment, take pain medication each day, and 67% of those men – 2 million people – take prescription pain drugs daily. This phenomenon sheds light on a key problem within the US labour market – even though unemployment is 4%, labour market participation has not rebounded, and is stuck at 62.9% – on a par with the 2014 rate, and lower than the 67% rate last seen in the late 1990s. The rate amongst prime–age men is particularly low – indeed, out of all OECD countries, only Italy has a lower rate. Mr Krueger argues that the use of opioid prescriptions must be addressed to increase particular of these men, highlighting that it affects labour participation by denting motivation and affecting individuals’ ability to pass drugs tests. (Financial Times, 7 September 2017)

➢Relatively little attention has been paid to private sector investment in global health R&D, with most measurements focusing on public sector expenditure. However, a study published by the Brookings Institute aimed to redress this by quantifying the return–seeking R&D investments in drugs, vaccines and therapeutics by the private sector, which includes pharmaceutical companies, venture capitalists and impact investments. The authors concentrated on overall R&D that focused on these areas, plus global health R&D that emphasizes medical treatments in the developing world, and neglected disease R&D, which focuses on drugs, vaccines and therapeutics for 35 specific illnesses which mainly affect low–income countries. Overall, they sound that at least US$ 159.9 billion is spent on health R&D annually, and that private sector investment accounts for US$ 5.9 billion of global health R&D in developing countries, mainly from pharmaceutical companies and venture capitalists. Private sector R&D on neglected diseases garners a mere US$ 511 million, mainly from private investors. Much of the growth in private global health R&D is from non–western companies, and looking ahead, the private sector will need to increase to counteract reductions in public health expenditure. The authors also make several recommendations for improving private investment, including creating viable markets, strengthening health governance and tax incentives. They also call for the funding gap between HIV, tuberculosis and malaria, which are relatively well–funded, despite having lower mortality rates than some non–communicable diseases, which affect more people but have less funding. (Brooking Institute, 12 September 2017)

➢China is one of the world’s largest providers of overseas aid, spending an estimated US$ 5 billion on aid. However, it has a reputation as a rogue donor – funding shoddy projects, mistreating workers coupled with low environmental standards. The benefits of China’s aid programme are largely kept secret, but a recent study by AidData looked at 4400 projects funded by China from 2000 to 2014. Their total value was US$ 350 billion, compared to the USA’s aid of US$ 424 billion. Unlike the USA, 20% of China’s aid is in grant form (US$75 billion – equivalent to the UK), with the rest being concessional lending at below–market interest rates, mainly to Chinese companies working overseas. Concessional lending fell out of favour in the 1990s as it overburdened recipients with debt. Despite this, AidData’s study, based on official announcements from Chinese commercial offices and from the finance and planning ministries of recipient countries, indicates that a 100% increase in China’s grant aid is associated with a 0.4% increase in recipient GDP after two years. This is in contrast to China’s concessional lending, which has no effect on the recipient’s GDP, and appears to consist of a subsidy to Chinese companies, alongside bribes to local elites. More positively, AidData’s study suggests that aid from China does not harm efforts from other donors. Overall, the study suggests that Chinese aid should focus more on grants, rather than loans. Second, Western agencies should co–operate more with China, to avoid duplication. And lastly, China should be more open about the successes of its overseas aid programme. (Economist, 12 October 2017).

➢Richard Thaler won the 2017 Nobel Prize in Economics, and his work on “nudge theory” – credited with helping people save more for retirement, eat more healthily and even with improving men’s aim into urinals – is based on the idea that small changes in people’s “choice architecture” can steer them towards beneficial decisions. However, nudge theory can also be used to steer people towards choices that work against them. Eg, Uber has developed techniques that push its drivers to work for longer – even at hours and locations that are less profitable for them – with drivers being shown their next fare before they’ve even dropped off their current passenger. In the UK, bookmakers advertise complex bets to gamblers (eg, whether a certain player will score first), which earns higher profit margins as gamblers consistently overestimate the probability of complex bets. These techniques have been termed “dark nudges”, and are becoming increasingly common in highly–complex and often poor value–for–money products, eg, sub–prime mortgages or complex mobile phone contracts. These approaches utilise nudge theory in ways that it was not intended, and consumers may respond by tuning out all nudges, including those which may be beneficial. Mr Thaler himself urges consumers to be vigilant, read the fine print, and give their business to companies that nudge for good, not bad. (MarketWatch, 20 October 2017)

## Energy

The Irish government is discussing projects with Brussels to improve its energy security post–Brexit. All of Ireland's imported gas and electricity flow via the UK, and Ireland is seeking to reduce its reliance on the UK following the UK’s departure from the EU. The talks with Brussels are covering potential projects such as a 1 billion Euro (US$ 1.65 billion) French electricity link, and a 500 million Euro (US$ 582 million) liquified natural gas terminal, for which Ireland is seeking EU financial support. The EU has already awarded 4 million Euros (US$ 4.66 million) for preparatory work on the proposed “Celtic Interconnector”, which will carry 700 MW of electricity between northwest France and southern Ireland. The EU may also support a liquid natural gas terminal on Ireland's west coast, which could be an attractive entry point to northern Europe for booming US gas exports. Peter O’Shea, from ESB, Ireland's power group, said that both the UK and Ireland have a mutual interest in ensuring smooth and low–cost energy flows between the UK and EU. “Ireland, north and south, Great Britain and Europe are all net importers of energy and have critical dependencies on each other. I would hope that pragmatism will prevail,” he said. (Power Engineering International, 27 July 2017)

➢Until recently, economists believed that power generated from offshore wind was an expensive and inefficient way of reducing carbon emissions, but in September 2017 this was blown apart by a massive fall in the cost of offshore wind in an UK government auction. This has led to suggestions that over the next 50 North Sea wind could be as important to the UK, as North Sea oil and gas were in the previous 50 years. Although wind power only accounts for 5% of Britain’s energy production, the auction showed how a commitment to wind power can lower its costs to the point where it can compete with gas and nuclear energy. Technological improvements in the industry have also improved its efficiency, and the British supply–chain is catching up by producing more wind turbines. The reformed auction system also promotes competition by allowing companies to decide where they should locate their offshore wind farms, rather than governments. All this bolsters the UK’s position as the world leader in off–shore energy, with capacity expected to double by 2020, and investment is tipped to reach US$ 11.5 billion in 2017–21 – more than expenditure on broadband infrastructure. However, pressure on suppliers to cut costs risks sloppy workmanship and delays. (Economist, 14 September 2017)

➢The goal of the entire world’s population having access to modern, reliable affordable energy by 2030 will not be achieved without full funding, according to Sustainable Energy for All (SEforALL), a body set up by the UN. SEforALL believes that this slow progress undermines effort to end poverty, food shortages and curb climate change, and denies people “a fundamental building block” of prosperity, and that the lack of funding to end the use of polluting fuels for cooking is “shocking”. Eg, nearly 90% of Bangladeshi people lack access to clean cooking facilities. Across the 20 countries with the biggest gaps in access, overall investment in domestic clean cooking energy resources averaged US$ 32 million a year, against the estimated annual need of US$ 4.4 billion. Rachel Kyte, the CEO of SEforALL, says that not enough governments – particularly in sub–Saharan Africa – prioritise clean cooking, and there is too much focus on designing more efficient stoves at the expense of providing clean fuels, such as gas and solar induction. However, there are notable exceptions, eg, Kenya, where the increasing use of mobile payments has attracted private–sector investment in “pay–as–you–go” solar energy systems. (Thomson Reuters, 18 September 2017)

➢Michael Bloomberg, a UN special envoy on climate change, has extended his US$ 164 million campaign against coal–burning from the USA to Europe, with US$ 50 million allocated to Europe. He has further plans to expand his campaign to the rest of the world. The funding will be used to support grassroots campaigns, research on the health impacts of coal, and legal action against coal plants that are breaking pollution laws. Mr Bloomberg is a prominent opponent to US President Trump’s stance on climate change. Coal accounts for 20% of the EU’s carbon emissions – Germany and Poland are the main emitters – and Mr Bloomberg aims to accelerate the decline in coal by capitalising on falling prices for renewal energy and rising concerns over air pollution. He credits the mass closure of US coal–fired power stations to civil society advocacy combined with falling costs of renewable energy. He is now looking for partners to extend his campaign into Asia, which burns coal on a large scale. However, he rejects arguments that coal plants are a fast and cost–effective way to bring electricity to the millions of people world–wide who lack access to electricity. “Number one, it is not the cheapest or quickest, and number two, how many people are you going to kill [with air pollution]”? he said. (The Guardian, 9 November 2017)

➢On 16 October, China’s main planning agency issued a notice to local authorities and state petroleum producers in northern cities, warning that gas supplies will be “insufficient” during the peak demand periods of the heating season – mid–November to mid–March. 2017 saw strong economic growth in China, accelerating gas consumption, and a cold winter will worsen shortages. State petroleum companies were directed to increase gas production and work on pipelines, as well as accelerate gas storage projects and import infrastructure. Gas is a cleaner fuel than coal, and switching from coal to gas for domestic heating will improve China’s air quality, but shortages may hamper the government’s efforts. The government has raising distribution prices to commercial users to prioritise domestic users, and China’s imports of liquid natural gas in September were the second–highest on record. The government has ordered an end to all coal–fired heating in northern cities, leading to a crash programme to scrap coal–fired boilers and build new distribution networks for natural gas. Despite these efforts, air pollution worsened over the Beijing–Tianjin–Hebei region, as concentrations of fine–smog forming particles rose by 10% from the previous year, according to the Ministry of Environmental Protection. (Radio Free Asia, 13 November 2017)

## Environment

➢China’s ban on importing garbage from overseas will take effect at the end of 2017, covering materials such as waste plastic and paper, as well as slag from steelmaking, and waste wool, ash, cotton and yarn. China is a major importer of waste – in 2016, it imported 7.3 million tonnes of waste plastics, valued at US$ 3.7 billion. In its notification to the World Trade Organization of the forthcoming ban, China cited the large amount of dirty or hazardous wastes that were mixed into the solid waste, and were causing serious problems with environmental pollution. China’s rapid industrial development has led to it struggling with waste disposal, leading to toxic waterways and cities blanketed in smog. It plans to conduct a nation–wide survey of pollution sources, and has urged local authorities to move quickly by launching local investigations. (Reuters, 18 July 2017)

➢Cobalt is a critical part of the lithium–ion batteries used in electric cars, and these batteries account for 42% of the global consumption of cobalt. This will increase further – some analysts predict a 30–fold increase by 2030 – as the world moves away from diesel and petrol cars towards electric cars. 60% of the world’s cobalt supplies is mined in the Democratic Republic of Congo (DRC). However, thus far, the average Congolese citizen – who are amongst the poorest people on Earth – has gained little from their country’s abundant supply of the metal. Indeed, according to a recent report from Global Witness, 30% the revenues paid to state bodies by mining companies – US$ 750 million – have disappeared. The west has poured in billions of US$ to the DRC in peace–keeping and aid, but foreign mining companies have extracted much more in gold, diamonds, tin, coltan, copper and cobalt. It is incredibly complex to ascertain if cobalt entering the supply chain was sourced without labour exploitation, violence and toxic pollution – and suppliers who boycott Congolese cobalt often ultimately harm small–scale artisan miners – but the same ingenuity which invented the electric car should be capable of solving the dilemma of powering them ethically. (Financial Times, 26 July 2017)

➢South Asia is home to 20% of the world’s population, and could see humid heat levels increase to unsurvivable levels by the end of the century unless action is taken. According to a study published in *Science Advances*, the region could experience summer heat waves with levels of heat and humidity beyond what humans can survive without protection. The study is the first to look at “wet–bulb temperature”, which combines temperate, humidity and the body’s ability to cool down, and the survivability threshold is considered to be 35°C. The study examined two scenarios, the “business–as–usual” scenario, where little is done to combat climate change, and the second scenario in which temperate change is kept to under 2°C, as pledged by the 2015 Paris accord. Under the “business–as–usual” scenario, wet–bulb temperatures are expected to approach the survivability threshold over most of South Asia, and exceed it in a few areas by 2100, affecting 30% of the region’s population. Farm workers would be worst affected as they have fewer opportunities to escape into air–conditioned environments. However, if global warming can be limited to 2°C, the population exposed to dangerous wet–bulb temperatures would increase from 0% to 2% – less than the 30% under the first scenario – and although temperatures would still reach 31°C, this is still below the fatal thresholder. Lead author Elfatih Eltahirhe, professor of environmental engineering at the Massachusetts Institute of Technology, calls for mitigation of the effects of climate change. “With mitigation, we hope we will be able to avoid these severe projections. This is not something that is unavoidable,” he said. (Jakarta Post, 3 August 2017)

➢According to a study published in the *Lancet*, global pollution contributes to an estimated 9 million deaths each year – around 1–in–6 of all deaths, whether through dirty air, tainted water, toxic industry etc. If correct, this means that pollution kills three times more people than HIV/AIDS, malaria and TB combined – and most of these deaths occur in developing countries. It found that poor air quality was the main driver, causing heart disease, strokes, lung cancer and other respiratory problems. The largest number of deaths were found in India and China, with an estimated 2.5 million and 1.8 million deaths respectively, and overall the authors estimated that pollution–related health problems and deaths cost 1.3% of developing countries’ GDP, compared to 0.5% in developed countries. According to Gina McCarthy, a former administrator at the US’s Environmental Protection Agency, addressing this problem is vital to moving people out of poverty, but that climate change would serve to worsen it. Philip Landigan, one of the study authors, states that poor developing countries can and should do more to reduce pollution, and would reap economic benefits from doing so. He also called for developed countries and aid foundations to support developing countries in reducing pollution. (Washington Post, 19 October 2017)

➢A new study used satellite data to track global artificially–lit surfaces, and found that these surfaces are increasing and growing brighter, producing more light pollution at night. In the second half of the 20th century, outdoor lighting grew by 3%–6% each year, due to electric lights. Whilst this has benefitted human productivity and safety, it means that nights are no longer dark enough, with 50% of Europe and 25% of North America experiencing modified light–dark cycles. Light pollution can have serious consequences for organisms, which have evolved in accordance with natural day–night cycles – additional, artificial, light is a stressor, and many organisms have not had enough time to adapt. 30% of vertebrates and more than 60% of invertebrates are nocturnal, and could affect interactions between species. Humans are also affected by artificial light, because certain physiological processes happen in the daytime, and others at night–time, and they often work against each other (eg, shift–workers who work against their biological day–night clocks can experience a range of health problems). The introduction of cheaper LED lighting has led to more energy devoted to lighting, as costs have fallen, and the blue light in LEDS is particularly disruptive for nocturnal patterns. Solutions to the growing problem of light pollution includes using LEDs without a blue component, and positioning light sources so they are not as bright but still effective (eg, dim, closely–spaced lights are more effective than bright lights spread out). (Seattle Times, 22 November 2017)

## Food, Water and Sanitation

➢The Toilet Board Coalition and Pune Municipal Corporation, India, have announced a collaboration aimed at making Pune the world’s first smart sanitation city. The Toilet Board Coalition, a business–led public–private partnership, and the city’s governing body, confirmed that they will develop smart, sustainable and resilient sanitation systems, delivered via the marketplace. The project will launch in January 2018, and its three work streams consists of: community toilets, focusing on optimising their usage and behavioural change; waste management and resource recovery; and exploring the use of sensors in the sanitation system for data capture. This is part of Prime Minister Modi’s Smart Cities campaign, and Pune has already been selected as one of Mr Modi’s “Smart Cities, and has also declared itself an official open–defecation–free city (the Indian government aims to eradicate open defecation by 2019). “Sanitation solutions that improve lives and make our citizens proud is at the centre of our project – working with global and local businesses and leveraging smart technologies,” said Prerna Deshbhratar of the Pune Municipal Corporation. (Cities Today, 30 August 2017)

➢The majority of New York school students are poor, with 75% qualifying for free or reduced price lunches. However, many children skip lunch rather than admitting that their families cannot afford to pay for it, and the national practice of “lunch shaming” – holding children accountable for unpaid lunch bills – has attracted attention. In light of this, New York City will make all school lunches free of charge, joining cities such as Boston, Chicago, Detroit and Dallas. The city’s administration does not expect to spend more on lunches as a result, as the city qualifies for a federal programme that pays for universal free lunches. New York City schools had already provided breakfast, and the city’s stand–alone middle–schools have had a free lunch scheme in place that provides lunch to an additional 10 000 children who would not necessarily have qualified for free school meals. (New York Times, 6 September 2017)

➢According to the Food and Agriculture Organization (FAO), for the first time in several years, the estimated number of undernourished people has increased, rather than decreased, to reach 800 million people worldwide. This means that the world has much more to do to reach the SDG of ending world hunger by 2030. Food insecurity is also high, and there is little evidence of improvement over the past decade. In the poorest countries, agricultural productivity is very low, with little improvement – and yields are generally lower for smallholder farmers where hunger is concentrated. The situation is worsened by a lack of private credit amongst smallholders, which hampers investment. Current agricultural policy is doing little to alleviate undernourishment, with increasing numbers of non–tariff barriers on agricultural trade, and agricultural subsidies indicate that money is being spent inefficiently (although developed countries have pledged to eliminate subsidies on agricultural exports). Moreover, China, which is becoming a major player in world food markets, is increasing its agricultural subsidies to match the entire OECD. In many countries, total spending on food and nutrition is US$ 10 per person/y, which is insufficient to reduce hunger, and food prices, which are starting to fall, may cause donors to shift attention elsewhere. Significant improvements in data, policies, resources and institutional performance are all required to end hunger by 2030. (Brookings, 23 October 2017)

➢Mr Aliko Dangote, Africa’s richest man with a net worth of US$ 13.7 billion, has pledged to invest US$ 100 million via his Dangote Foundation to tackle malnutrition in Nigeria’s worst–affected areas. It aims to reduce the prevalence of under–nutrition by 60% in these areas, specifically the northeast and northwest of the country. Mr Dangote set up the Dangote Foundation in 1993, which invests in health, education, economic empowerment and disaster relief. The announcement was made at the Global Nutrition Summit, which aims to accelerate the global response to malnutrition. “Nigeria’s high malnutrition rate is undermining progress towards improving child health and putting the brakes on economic development. By investing in nutrition, we aim to directly improve the lives of Nigerian families and to empower our citizens to reach their full potential,” said Ms Zouera Youssoufou, the Managing Director and CEO and Dangote Foundation. (Forbes, 6 November 2017)

➢Cambodia is lauded as one of the most successful countries in getting people to install and use toilets – and key to that success is a peer–pressure strategy developed in Bangladesh and adopted across the world. Pressure from the community inhibits people from defecating in the open, and they are encouraged to use the toilets of friends or family instead, if they lack one of their own. Mr Phuy Seakphy has developed a thriving toilet installation business – the toilets consist of simple concrete pipes sunk into the ground, and he now sells 15–20 toilets a month. Women and girls risk attack if they go into fields at night to relieve themselves. The shortage of toilets in rural Cambodia also has a significant effect on children, with an estimated 33% of children aged under 5 years suffering from stunted growth. “When children are exposed to faeces in their environment, they repeatedly get bouts of diarrhoea. So the good things that are going in are coming straight out,” says James Wicken, the country director of WaterAid Cambodia. (Al Jazeera, 20 November 2017)

## Peace and Human Rights

➢Iran has one of the highest rates of capital punishment in the world – executing at least 567 people in 2016 and nearly 1000 in 2015 – but its lawmakers have proposed changes to Iranian anti–drugs legislation which may abolish the death penalty for certain drug–related crimes, mainly petty, non–violent crimes. The government has faces pressure to reform these laws, as critics highlight that they have done little to deter drug–related crime in a country that is a major transition route for drugs smuggled from Afghanistan. Currently, 70% of all Iranian executions are for drug–related crimes, and can be applied for the trafficking or possession of 30g of heroin or cocaine. In November 2015, Hassan Nowruzi, Iran’s parliamentary judicial spokesman, called for reform, revealing that 5000 people were on death row for drugs–related offences – the majority being first–time offenders aged 20–30 years. There are an estimated 2000 Afghan people in Iran’s prisons on drug–smuggling charges, and there are concerns that many do not receive fair trials and lack access to defence lawyers. The campaigning group Human Rights Watch has called for a moratorium on the Iranian death penalty whilst the reforms are debated – “it makes no sense for Iran’s judiciary to executive people now under a drug law that will likely bar such executions,” said Sarah Leah Whitson, the group’s Middle East Director. However, the proposed changes are opposed by hardliners in the country’s judiciary, who defend their “tough stance” on the issue. “In some cases, including drug trafficking, we’re forced to act quickly, openly and decisively” said Ayatollah Sadegh Larijani, Iran’s judiciary head. (Radio Free Europe, 23 July 2017)

➢In the month following the disappearance of Mr Santiago Maldonado, an activist for the rights of indigenous peoples, thousands of Argentinians have demonstrated across the country, demanding answers to his disappearance. Mr Maldonado was last seen when border police evicted a group of indigenous Mapuche people from lands in Patagonia owned by the clothing company Benetton. Mr Maldonado’s disappearance is a reminder of the estimated 30 000 people who died or disappeared during Argentina’s 1976–1983 military dictatorship. According to witnesses, Mr Maldonado was taken alive, and government investigations have not uncovered any information on his whereabouts. The UN, Amnesty International and Human Rights Watch have said that Mr Maldonado’s disappearance requires urgent action from the Argentine President, Mauricio Macri. (Al Jazeera, 2 September 2017)

➢Cambodia’s Prime Minister Hun Sen’s governing party (the Cambodian People’s Party, or CPP) has faced widespread condemnation over its actions ahead of general elections scheduled for July 2018. These have ranged from targeting the opposition Cambodia National Rescue Party (CNRP), as well as orchestrating the closure of independent media outlets and restricting NGOs. On 16 November, Cambodia’s Supreme Court unanimously ruled that the CNRP be dissolved for its part in plotting a “coup” against the government, essentially eliminating any opposition to the government. The CNRP leader, Kem Sokha, is under arrest for allegedly collaborating with the US to overthrow the ruling CPP – charges which the US embassy has rejected. In response, international donors (including the EU and Sweden) are withdrawing aid from the country, or are threatening to do so, if restrictions are not lifted. Critics argue the government’s actions threaten Cambodia’s fragile democracy and call the legitimacy of the 2018 election into question. However, Prime Minister Hun Sen remains firm, saying that he would “welcome” any withdrawal of aid, and that Cambodia will rely on assistance from China, one of its few allies. (Radio Free Asia, 21 November 2017)

➢The 2017 Ibrahim Index of African Development shows that the rate of progress on human development has slowed over the past 5 years, with progress in education nearly “grinding to a halt”, and the public is losing faith in governments’ ability across the continent to improve health services. The index measures human development, based on 26 indicators focusing on welfare, education and health, ranking countries and reporting on their progress. Lower commodity prices could have led to budget cuts that may have affected progress, but there is also a risk from complacency following Africa’s gains over the past 10 years. Another factor may be the transition from the Millennium Development Goals, which prioritised education and health, to the Sustainable Development Goals, whose wider and more complex objectives may be more difficult to implement. The faltering progress in education is particularly concerning in a region where 41% of the population is aged under 15. Côte D'Ivoire, Egypt and Togo have led Africa in human development progress, with Côte D'Ivoire moving into a period of stability after the end of conflict, and Egypt also stabilising after the Arab Spring in 2014 and improving its provision of basic health services (although its human rights and citizen participation indices have declined). Togo has improved access to antiretroviral treatment, although there are street protests about the transfer of power from the former president to his son. Libya, Ghana and Sierra Leone experienced the greatest deteriorations in the human development index; Libya due to shortages of social safety nets, the Ghanaian public is losing confidence in their government’s ability to handle basic health services, and Sierra Leone’s efforts to narrow income gaps are losing momentum. Overall, Mauritius, Seychelles and Botswana are leading the continent’s development index, whilst Somalia, South Sudan and the Central African Republic languish at the bottom. However, more hopefully, the index found that antiretroviral treatment is the most improved indicator. In 2000, less than 1% of people in need of treatment received it, but in 2016, 54% received it. (Devex, 20 November 2017)

➢Yemen has long since lost its title of *Arabia Felix* (“Fortunate Arabia”), suffering from civil wars, tribalism, jihadist violence and appalling poverty – but the ongoing war between a Saudi–led coalition and the Houthis, a Shia militia backed by Iran is the worst yet. It is on the brink of famine, crises over waste disposal, sewerage system and water supplies have led to the worst cholera outbreak in recent history – and the UN estimates that 75% of its 28 million people need humanitarian aid. So far, the outside world has paid little attention, perhaps because it is inured after years of bloodshed in the Middle East – or, more cynically, because Yemen is further from Syria than Europe so Yemenis do not generally seek asylum there. Even setting aside humanitarian considerations, the world cannot afford another failed state that becomes a breeding–ground for terrorism. The war has its roots in the 2011 Arab Spring, when mass unrest forced the president, Mr Ali Abdullah Saleh to step down, but his successor, and the resulting constitutional changes, were rejected by the Houthi rebels, who allied themselves with the former president. The war has dragged on – the Houthis are too weak to rule over Yemen but as a well–entrenched militia, too powerful for Saudi Arabia to defeat. UN–led peace talks have led with the demand that the Houthis surrender, which is unrealistic. A more pragmatic approach would be to freeze the conflict, and find another mediator, such as Oman or Kuwait. A deal should involve the phased withdrawal of Houthi fighters from key areas, and the end to the Saudi blockade. Yemen needs an inclusive government, elections, and a new state structure, and Saudi Arabia will need guarantees that Iranian arms are not flowing into Yemen. (Economist, 30 November 2017)

## Science and Technology

➢Researchers from New York University are beginning to recruit participants into “The Human Project”, which will enroll 10 000 people to share a trove of personal information, including cellphone locations, credit card swipes, blood samples and life–changing events – over the next 20 years. The project will channel the data streams to build a picture of health, ageing, education and other aspects of human life. Although the “Human Project” is one of many “big data” health studies, its approach on the scope of data it plans to simultaneously collect is unique. Participants will be invited to join, following demographically–representative groupings, and will be screened for blood groups, genetics and IQ, and will give access to medical, financial and educational records, plus cellphone data. They will be given activity trackers, weighing scales and surveys, and will have follow–up tests at 3 years. The project team hope that the results will illuminate the linkages between health, behaviour and circumstances, and will shed light on conditions from asthma to Alzheimer disease. The data will be subject to stringent protection measures, with researchers safeguarding data from all eventualities apart from major terrorism probes. (STAT news, 19 June 2017)

➢The World Health Organization estimates that there are 2–5 million people with missing limbs in the developing world, many of whom do not have prosthetic limbs. To help overcome this, Ashraf Mizo, a Sudanese engineering student, is working on simplified and inexpensive prosthetic limbs through 3–D printing technology and machine learning. Until recently, 3–D printing has been viewed as far too expensive for prosthetic limbs, and that prosthetic limbs are regardless too complex for 3–D printing. However, Mr Mizo uses a simplified calibration system and a reduced number of actions –open, close, pinch and grip – that enabled him to print a prosthetic arm for US$ 20. Although the prototype has not yet been tested on amputees, Mr Mizo plans to partner with an amputee organisation in Sudan to start fitting prosthetic hands onto patients. (Forbes, 31 August 2017)

➢In the USA, radiologists on average earn US$ 400 000 a year – nearly double the salary of a family doctor – but many radiologists are concerned over job security. MRI and CT scans are now routine procedures, and their data can be stored digitally, so radiologists have to assess many more images – a radiologist may review 20–100 scans a day, and each scan can have thousands of images. This makes radiology particularly amenable to artificial intelligence and machine learning, as it is based on humans examining pictures and discerning patterns amongst the images – which computers can excel at. Technological companies, such as IBM, Google and GE are developing technologies that uses machine learning – training a computer by feeding it thousands of images – that may lead to an algorithm that can diagnose heart disease or strokes more quickly and cheaply than a human. These algorithms could also potentially check a single scan for multiple diseases. However, Dr John Mongan of UCSF is unconcerned about radiologists being replaced by artificial intelligence in the longer–term; he believes that their role will evolve to spend less time examining images and more time selecting algorithms and interpreting results. If radiologists can work with artificial intelligence, they may benefit from it, rather than be replaced by it. (NPR, 4 September 2017)

➢The 2017 Nobel Prize in Physiology and Medicine was awarded to Jeffrey C Hall, Michael Rosbash and Michael W Young for their work on the molecular mechanisms that control the body’s circadian rhythms. They used a gene isolated from fruit flies to determine how it encoded for a protein that accumulated in cells at night, and then degraded during the day. Their research casts light on the biology of humans and other multicellular organisms whose biological clocks function on the same principle of 24–hour rhythms of sleep and wakefulness, plus functions such as blood pressure, heart rate, alertness, temperature and reaction times. The Nobel committee judged that their work was pivotal, because understanding that misalignment between a person’s lifestyle and the rhythm dictated by an inner time–keeper could affect well–being and could contribute to the risks for various diseases. (New York Times, 2 November 2017)

➢South Africa has the highest incidence of HIV in the world, but of the 7 million people infected with HIV, only 50% are receiving treatment, and the country's public health system is being pushed to breaking–point. It is hoped to bring HIV under control by doubling the number of people receiving treatment, but the health system cannot afford to double the number of doctors, nurses and pharmacists. To cope with demand, the health system is making more use of technology, including robots. Eg, the Helen Joseph hospital, South Africa's busiest HIV clinic, uses a robot pharmacist to collect and package drugs; and although its work is checked by humans, overall waiting times at the clinic have fallen from 4 hours to under 20 minutes. Researchers are now working on ATM–style medication dispensaries which will be even swifter. Moreover, Africa as a whole makes up 66% of the world's pregnancy and childbirth–related deaths, despite having one–sixth of the world's population – and 1–in–9 children dies before their 5th birthday. Many of these deaths could be avoided by simple diagnostic tools, and new technologies are improving this. For instance, the electronics company Phillips have developed a wind–up ultrasound machine that requires no electricity supply and provides a digital printout of a foetus's heartbeat, that more accurately identifies urgent cases than the trumpet horn which is normally used. Phillips have also developed an easily–transportable solar–powered chest monitor that measures a baby's rate of breathing to help diagnose pneumonia. Companies such as IBM are using artificial intelligence to determine how drug–resistant TB spreads, and how genes that protect against malaria contribute to an increased risk of certain cancers. Mobile phone apps are being used to train health workers, and Médecins Sans Frontières is experimenting with a smartphone to determine if a cough indicates asthma or pneumonia. According to Solomon Assefa, the head of IBM's research in Africa, the continent's shortage of health professionals is forcing it to experiment with technology. High–income countries are not yet under the same pressure, but ageing populations and the increasing burden of chronic diseases may lead them to learn from Africa's experiences with technology. (Economist, 9 November 2017)

We are grateful to Jelena Bućan for her contribution towards the News articles in this volume.

